# Precision of a new ocular biometer in children and comparison with IOLMaster

**DOI:** 10.1038/s41598-018-19605-6

**Published:** 2018-01-22

**Authors:** Xinxin Yu, Hao Chen, Giacomo Savini, Qianqian Zheng, Benhao Song, Ruixue Tu, Jinhai Huang, Qinmei Wang

**Affiliations:** 1 School of Ophthalmology and Eye Hospital, Wenzhou Medical University, Wenzhou, Zhejiang, China; 2 G.B. Bietti Foundation IRCCS, Rome, Italy

## Abstract

To assess the repeatability and reproducibility of AL-Scan in agreement with those by the IOLMaster in healthy children, two skilled operators measured ocular parameters in 58 children. The parameters included keratometry (K) values, anterior chamber depth (ACD), axial length (AL), central corneal thickness (CCT), pupil diameter (PD), and corneal diameter (CD). The cohort comprised of 32 boys and 26 girls. The AL-Scan measurements showed high repeatability, as the test-retest repeatability (TRT) values of AL, CCT, ACD, Kf, Ks, Km, CD, and PD were 0.09 mm, 5.1 μm, 0.04 mm, 0.28 D, 0.24 D, 0.21 D, 0.39 mm, and 0.22 mm, respectively. The within-subject coefficient of variation (CoV) was low (<0.35%) and the intraclass correlation coefficients (ICC) of all parameters were >0.85. The interobserver reproducibility was excellent with low values of TRT and ICC > 0.95. The CoV of AL, CCT, ACD, and K was <0.22%. The 95% limits of agreement between the AL-Scan and the IOLMaster were narrow for all parameters except for CD. The repeatability and reproducibility of the new biometer, Al-Scan, was excellent for all parameters and can be routinely used in children to measure the biometric values.

## Introduction

Myopia is a refractive error of eyes that occurs globally, causing different degrees of vision impairment. It is speculated that myopia and high myopia will show a significant increase in prevalence worldwide, affecting nearly 5 billion and 1 billion individuals, respectively, by 2050^1^. Hitherto, the prevalence of myopia is 70–87% in Asian schoolchildren and young adults^2,3^. Therefore, many studies have focused on myopia in children. Axial length (AL) exerts a major effect on myopic progression^4^. Accurate and precise ocular biometric parameters are crucial for studying the pediatric myopia. Both ultrasonic and optical methods can obtain ocular biometric parameters. Although ultrasonography is the conventional measuring method, it might cause discomfort in patients, corneal epithelial defect, or infection^5–9^. Unlike ultrasound biometry, optical biometry devices have the advantage of not requiring any contact with the eye. The first non-contact optical biometer (IOLMaster, Carl Zeiss Meditec AG, Germany) was introduced in 1999^10–12^, followed by an increasing number of optical biometers that prevailed commercially. A new optical biometer (AL-Scan, Nidek Co., Ltd., Japan) was introduced recently^10,13^. In a single measurement, it can obtain the ocular biometric data, including keratometry (K) values, anterior chamber depth (ACD), AL, central corneal thickness (CCT), pupil diameter (PD), and corneal diameter (CD) based on partial coherence interferometer (PCI) and Scheimpflug imaging techniques^14,15^. Some studies on the AL-Scan have been carried out in different populations; however, no study has yet evaluated the precision of its measurements in a pediatric population. Thus, the present study assessed the intraobserver repeatability and interobserver reproducibility of the measurements by AL-Scan in children, and compared the differences between the AL-Scan and IOLMaster, which is the gold standard for biometric measurement.

## Results

A total of 58 right eyes from 58 healthy children were included in the present study. The cohort comprised of 32 boys and 26 girls with the mean age of 8.4 ± 1.52 (range: 6–14) years.

### Intraobserver Repeatability

Table 1 shows the intraobserver repeatability outcomes for biometric measurements obtained using the AL-Scan. The results indicated high intraobserver repeatability, with the TRT values of AL, CCT, ACD, Kf, Ks, Km, CD, and PD less than 0.09 mm, 5.01 μm, 0.04 mm, 0.28 D, 0.24 D, 0.21 D, 0.39 mm, and 0.22 mm, respectively. The ICC was > 0.90 for all measurements except for CD. The CoV of most parameters was < 0.35%; the highest CoV (>8%) was observed for the astigmatism magnitude.

**Table 1:** Intraobserver repeatability outcomes for biometric measurements obtained using AL-Scan partial coherence interferometry in children.

Parameter	observer	Mean ± SD	Sw	TRT	CoV (%)	ICC
AL (mm)	1st	23.83 ± 1.22	0.03	0.09	0.14	0.999
	2nd	23.82 ± 1.22	0.03	0.09	0.14	0.999
CCT (μm)	1st	547.40 ± 31.97	1.81	5.01	0.33	0.997
	2nd	547.42 ± 31.98	1.79	4.95	0.33	0.997
ACD (mm)	1st	3.81 ± 0.20	0.01	0.04	0.34	0.995
	2nd	3.82 ± 0.20	0.01	0.03	0.30	0.997
Kf (D)	1st	43.76 ± 1.54	0.10	0.27	0.23	0.996
	2nd	43.77 ± 1.54	0.10	0.28	0.23	0.997
Ks (D)	1st	42.47 ± 1.43	0.09	0.24	0.20	0.996
	2nd	42.50 ± 1.44	0.08	0.23	0.19	0.996
Km (D)	1st	43.11 ± 1.44	0.08	0.21	0.17	0.997
	2nd	43.13 ± 1.44	0.08	0.21	0.18	0.997
Astigmatism	1st	1.28 ± 0.80	0.11	0.30	8.54	0.982
	2nd	1.28 ± 0.83	0.11	0.29	8.28	0.984
J_0_ (D)	1st	−0.61 ± 0.40	0.05	0.15	—	0.981
	2nd	−0.61 ± 0.41	0.06	0.15	—	0.982
J_45_ (D)	1st	0.06 ± 0.21	0.06	0.16	—	0.928
	2nd	0.06 ± 0.20	0.05	0.13	—	0.946
CD (mm)	1st	12.08 ± 0.35	0.14	0.38	1.12	0.862
	2nd	12.05 ± 0.35	0.14	0.39	1.15	0.859
PD	1st	7.94 ± 0.59	0.08	0.22	1.00	0.982
	2nd	7.96 ± 0.57	0.07	0.20	0.90	0.984

AL = axial length, CCT = central corneal thickness, ACD = anterior chamber depth, Kf = flattest keratometry, Ks = steepest keratometry, Km = mean keratometry, CD = corneal diameter, SD = standard deviation, Sw = within-subject standard deviation, TRT = test-retest repeatability (2.77 Sw), CoV = within-subject coefficient of variation, ICC = intraclass correlation coefficient.

### Interobserver Reproducibility

Table 2 shows interobserver reproducibility outcomes for biometric measurements obtained using the AL-Scan. All parameters revealed high reproducibility with low S_w_ values. The CoV of AL, CCT, ACD, and K values were lower than 0.22%; the highest CoV (6.68%) was observed for the astigmatism magnitude. The ICC values were>0.95 for all measurements.

**Table 2:** Interobserver reproducibility outcomes for biometric measurements obtained using AL-Scan partial coherence interferometry in children.

Parameter	Mean difference ± SD	Sw	TRT	CoV (%)	ICC
AL (mm)	0.01 ± 0.03	0.02	0.06	0.09	1.000
CCT (μm)	−0.02 ± 1.51	1.06	2.93	0.19	0.999
ACD (mm)	0.00 ± 0.01	0.01	0.02	0.22	0.998
Kf (D)	−0.02 ± 0.09	0.08	0.21	0.17	0.998
Ks (D)	−0.02 ± 0.11	0.06	0.18	0.15	0.998
Km (D)	−0.02 ± 0.08	0.06	0.16	0.13	0.998
Astigmatism	0.01 ± 0.12	0.09	0.24	6.68	0.989
J_0_ (D)	0.00 ± 0.06	0.04	0.11		0.990
J_45_ (D)	0.00 ± 0.05	0.03	0.09		0.976
CD (mm)	0.03 ± 0.10	0.07	0.21	0.62	0.955
PD	−0.01 ± 0.11	0.08	0.22	1.01	0.981

AL = axial length, CCT = central corneal thickness, ACD = anterior chamber depth, Kf = flattest keratometry, Ks = steepest keratometry, Km = mean keratometry, CD = corneal diameter, SD = standard deviation, Sw = within-subject standard deviation, TRT = test-retest repeatability (2.77 Sw), CoV = within-subject coefficient of variation, ICC = intraclass correlation coefficient.

### Agreement Between the AL-Scan and IOLMaster

**Figure 1:**
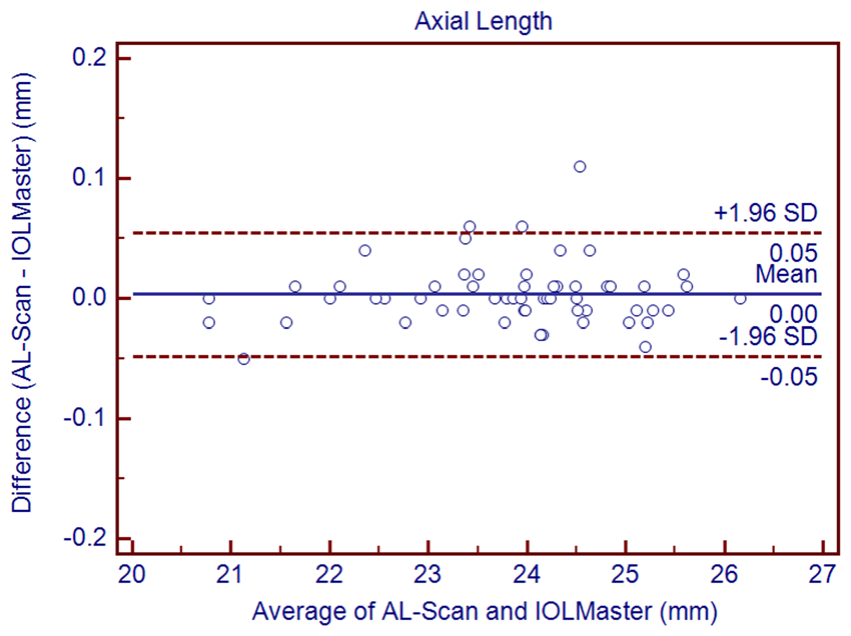
Bland–Altman graphs for pairwise comparisons between the AL-Scan and IOLMaster measuring the axial length in children. The mean difference and 95% limits of agreement are indicated as solid and dashed lines, respectively.

**Figure 2:**
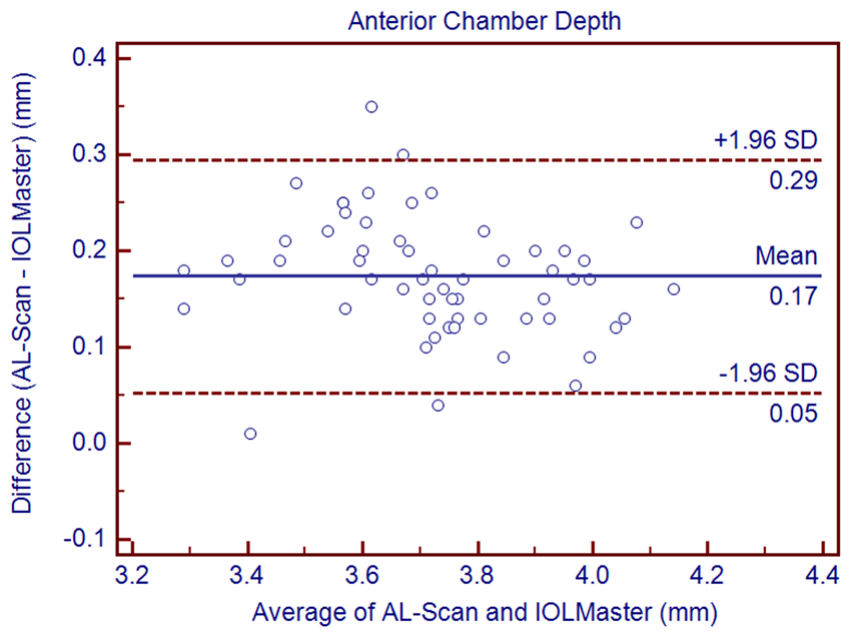
Bland–Altman graphs for pairwise comparisons between the AL-Scan and IOLMaster measuring the anterior chamber depth in children. The mean difference and 95% limits of agreement are indicated as solid and dashed lines, respectively.

**Figure 3:**
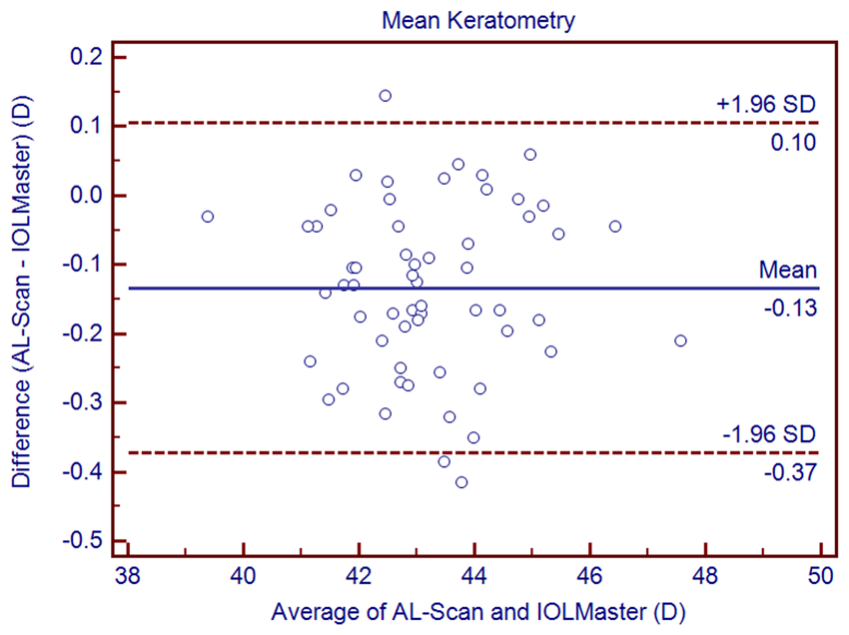
Bland–Altman graphs for pairwise comparisons between the AL-Scan and IOLMaster measuring the mean keratometry in children. The mean difference and 95% limits of agreement are indicated as solid and dashed lines, respectively.

**Figure 4:**
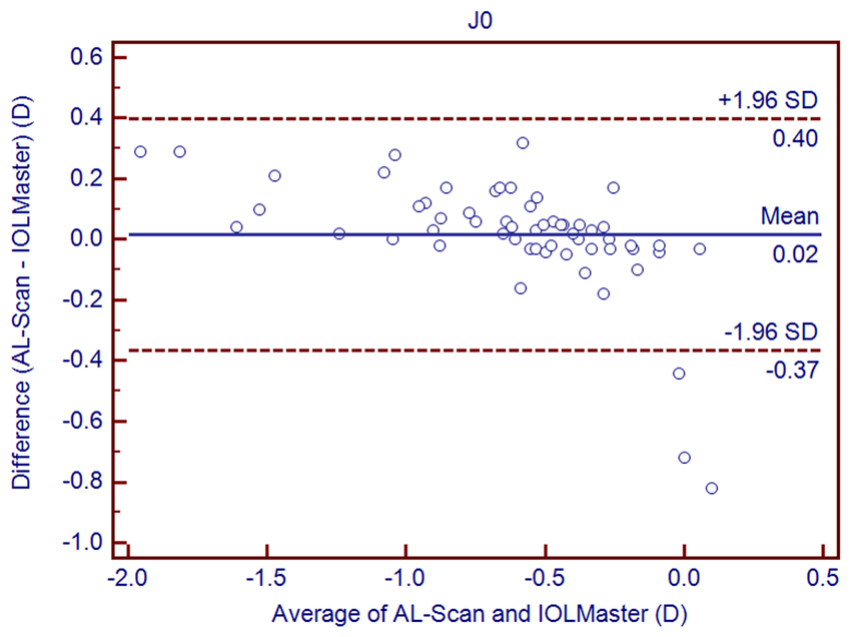
Bland–Altman graphs for pairwise comparisons between the AL-Scan and IOLMaster measuring J_0_ in children. The mean difference and 95% limits of agreement are indicated as solid and dashed lines, respectively.

**Figure 5:**
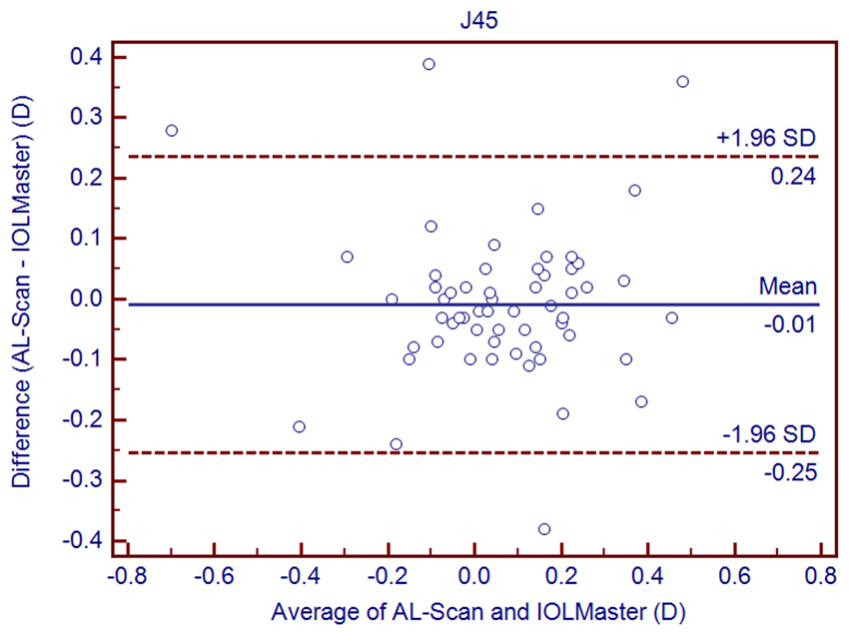
Bland–Altman graphs for pairwise comparisons between the AL-Scan and IOLMaster measuring J_45_ in children. The mean difference and 95% limits of agreement are indicated as solid and dashed lines, respectively.

**Figure 6:**
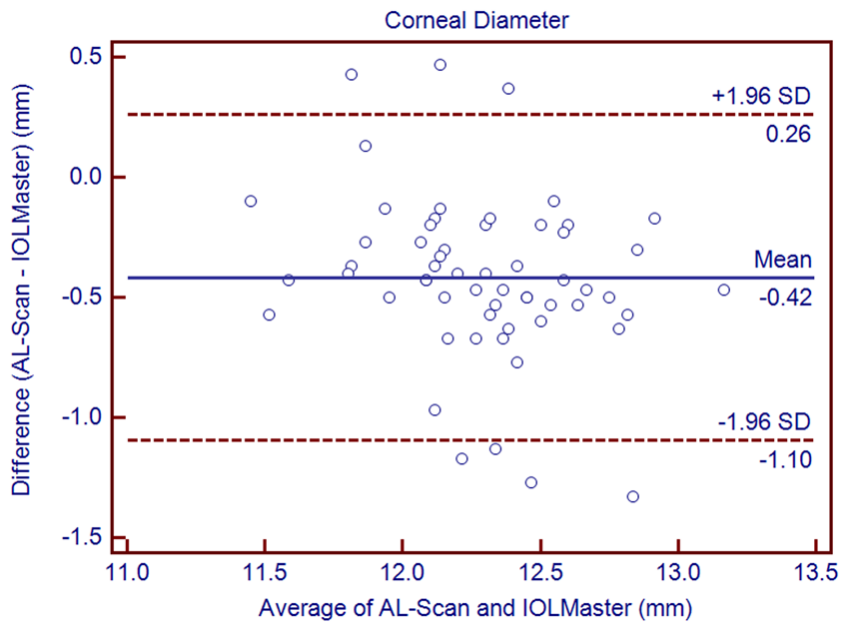
Bland–Altman graphs for pairwise comparisons between the AL-Scan and IOLMaster measuring the white-to-white in children. The mean difference and 95% limits of agreement are indicated as solid and dashed lines, respectively.

Table 3 shows the differences between the AL-Scan and IOLMaster. Statistically significant differences were observed between the AL-Scan and IOLMaster with respect to all the parameters except AL, J_0_, and J_45_. Although these differences were statistically significant, the 95% LoAs were narrow for all parameters except for CD, whose 95% LoAs were between −1.10 mm and 0.26 mm (Figs 1–6).

**Table 3:** The mean difference, paired T-test, and 95% limits of agreement (LoA) for differences between the AL-Scan and the IOLMaster partial coherence interferometry in children.

Device Pairings	Mean Difference ± SD	*P* Value	95% LoA
AL (mm)	0.00 ± 0.03	0.346	−0.05 to 0.05
ACD (mm)	0.17 ± 0.06	0.000	0.05 to 0.29
Kf (D)	−0.08 ± 0.14	0.000	−0.36 to 0.20
Ks (D)	−0.19 ± 0.17	0.000	−0.52 to 0.14
Km (D)	−0.13 ± 0.12	0.000	−0.37 to 0.10
Astigmatism	−0.11 ± 0.20	0.000	−0.50 to 0.27
J_0_ (D)	0.02 ± 0.19	0.537	−0.37 to 0.40
J_45_ (D)	−0.01 ± 0.12	0.608	−0.25 to 0.24
CD (mm)	−0.42 ± 0.35	0.000	−1.10 to 0.26

AL = axial length, ACD = anterior chamber depth, Kf = flattest keratometry, Ks = steepest keratometry, Km = mean keratometry, CD = corneal diameter, SD = standard deviation.

### Intraocular Lens Power Calculation

The calculated mean IOL power was 19.64 ± 3.20 D with the AL-Scan and 19.64 ± 3.19 D with the IOLMaster. The mean difference between the two biometers was 0.13 ± 0.14 D. The 95% LoAs were narrow (−0.15 to 0.41 D). The largest differences between the two biometers were 0.44 D.

## Discussion

AL is a major factor for the calculation of intraocular lens (IOL) power in pediatric cataract surgery. An AL difference of 1 mm leads to a refractive error of approximately 2.7–3.0 D^16,17^. In addition, estimating the progression of myopia also depends on accurate AL measurement. Sahin *et al.*^18^ used the Lenstar LS 900 (Haag-Streit AG, Koeniz, Switzerland) to acquire the AL of 23.22 ± 0.83 mm in school-age children. Our data with the AL-Scan (23.83 ± 1.22 mm) revealed little differences with respect to those reported with the Lenstar. Huang *et al.*^15^ found that the TRT of AL, as measured by the AL-Scan, was 0.05 mm in adults, thereby indicating excellent intraobserver repeatability. In the current study, the TRT of AL was low (0.09 mm); therefore, we speculate that the AL measured by the AL-Scan in children had excellent repeatability, similar to that in adults. In addition, the TRT of AL was 0.06 mm in reproducibility outcomes, which showed high interobserver reproducibility in a pediatric population.

Agreement between the AL-Scan and IOLMaster was high for the AL measurement with a mean difference of 0.00 ± 0.03 mm, and the 95% LoAs were between −0.05 and 0.05 mm. The mean difference was smaller than that observed in the study between the Lenstar and IOLMaster by Hoffer *et al*.^19^, who reported a mean difference of 0.03 mm. Jasvinder *et al.*^20^ also indicated that the results of Lenstar and IOLMaster were consistent in cataract patients (95% LoA was −0.04–0.07 mm) with respect to the current study. A few previous studies on cataract patients displayed a robust agreement between the AL-Scan and IOLMaster^10,15,21^. Therefore, we can consider that the AL-Scan and IOLMaster provided interchangeable AL measurements in a pediatric population. However, one of the major obstacles for ocular measurements in children is fixation. If the child does not stare at the target well, unreliable AL measurements may be obtained. The AL-Scan can provide a series of ocular parameters by a single measurement, thereby facilitating the cooperation of the children for reliable results.

Regarding ACD, a statistically significant difference with respect to the IOLMaster was found, with a relative higher mean value measured by the AL-Scan. However, a difference of 0.17 mm was not clinically relevant. This difference might be attributed to the usage of a lateral slit by IOLMaster to measure the ACD, whereas the AL-Scan is based on the Scheimpflug principle. Also, the IOLMaster and its previous versions have been reported to measure the lower ACD values than the other optical biometers in previous studies^10,22–24^.

The AL-Scan measures CCT using the Scheimpflug principle, whereas the IOLMaster does not acquire this parameter. The high ICC (0.997 or 0.999) and the low TRT showed that this instrument can provide highly repeatable and reproducible measurements of CCT. Moreover, the ICC values were higher than those in the study by Kola *et al*. and the TRT values were less than that in our previous study^9,15^. The study by Chen *et al.*^25^, Huang *et al.*^26^, and Savini *et al.*^27^ discovered that the ICC for the rotating Scheimpflug imaging was similar to the current study.

With low TRT (<0.28 D for repeatability and 0.21 D for reproducibility) and low CoV (<0.23% for repeatability and 0.17% for reproducibility), we found that AL-Scan provided highly repeatable and reproducible results for K values. The TRT of K values and astigmatism magnitude were <0.5 D, which was in the clinical range. In addition, similar results had been reported by Srivannaboon *et al.*^28^ in cataract patients. The 95% LoA showed high agreement in Kf and Ks between the AL-Scan and IOLMaster, although the mean Ks value was higher with the IOLMaster than that with the AL-Scan, which might induce slight selection bias in the calculation of IOL power, thereby necessitating constant optimization. Nevertheless, the agreement of corneal power and astigmatism was clinically acceptable. The reason for the difference could be attributed to the usage of a double-ring by AL-Scan to measure keratometry, which could provide more data than the IOLMaster. Moreover, the IOLMaster provided slightly steeper K values than the AL-Scan. Similar findings have been reported when the IOLMaster was compared to other devices such as Lenstar^29^.

Repeatability and reproducibility of CD and PD were high in children, thereby confirming the results previously reported with the same biometer by Kola *et al*. in adult patients^9^. The study revealed that the agreement for CD was not satisfactory, since the 95% LoA was between −1.10 and 0.26 mm. Both devices used the same principle to assess CD; however, the AL-Scan took advantage of the 525-nm LED measuring the CD that could avoid the blurred images caused by wide-spectrum laser. Although the consistency of the CD was not as satisfactory as other parameters, the 95% LoA values were narrower than those in the previous studies^15,21^. This might be due to the clearer corneal limbus in children than the elders suffering from cataract.

The present study has some limitations. First, we did not include the patients suffering from pathological myopia, which might influence the cooperation. Second, individuals with keratoconus eyes were not enrolled. These drawbacks necessitate future investigations considering these parameters.

In conclusion, we found that the new optical biometer presented excellent intraobserver repeatability and interobserver reproducibility for all parameters in children. Therefore, the AL-Scan can be routinely used in children to measure the biometric values. Moreover, the AL-Scan and IOLMaster were highly consistent with respect to a majority of the parameters.

## Methods

### Subjects

This prospective study consisted of normal children at the Eye Hospital of Wenzhou Medical University, Wenzhou, China. Children with ophthalmic surgery, dry eye, corneal disease, contact lens wear, strabismus, amblyopia, or ocular trauma were excluded. All eyes went through comprehensive ophthalmologic examinations before measurements, including subjective refraction, noncontact intraocular pressure measurement, slit-lamp microscopy examination, and ophthalmoscopy. The research was approved by the Board of Eye Hospital of Wenzhou Medical University, which adhered to the tenets of the Declaration of Helsinki. Informed consent was obtained from the childrens’ parents after explaining the purpose of the research.

### Instruments

The AL-Scan (software V.1.03) is based on PCI, measures the AL through an 830-nm infrared laser diode^12^. The optical biometer takes advantage of the light-emitting diode (LED) to assess the K values, CD, and PD^8^. The K values were measured at the 2.4 and 3.3 mm diameters^10^. In the current study, K values at the 2.4 mm diameters were recorded, as it is similar to that used by the IOLMaster. Then, the K values were calculated from the anterior corneal radius using the keratometric index of 1.3375, which included the flattest keratometry (Kf), the steepest keratometry (Ks), and the mean keratometry (Km). The measurement of CCT and ACD uses the Scheimpflug principle^9^. During a measurement, the AL-Scan performs in an automated mode measuring all the biometric variables.

Corneal astigmatism could be transformed into vector form, which included J_0_ and J_45_ based on the following formulae:

J_0_ = (-cylinder/2) cos (2 × axis)

J_45_ = (-cylinder/2) sin (2 × axis)

J_0_ refers to the cylinder at 90° and 180° meridians, whereas J_45_ refers to cylinder at 45° and 135° meridians. The axis is the flattest meridian.

The IOLMaster (version 5.4) is based on dual-beam PCI principle and utilizes a 780-nm infrared laser diode to measure the AL^10^. The ACD, defined as the length between the corneal epithelium and the anterior surface of the lens, is measured by lateral slit illumination^15^. The K values were calculated by analyzing the data from a hexagonal array of 6 points reflected off the surface of the cornea in the optical zone of an approximately 2.5 mm diameter^10,30,31^. CD was assessed by 3 consecutive measurements.

### Measurement Technique

Children were measured in a random order in order to avoid the methodological bias. 1% cyclopentolate hydrochloride eye drops were used (1 drop every 5 minutes, 3 times). After 40–60 min, the disappearance of the light reflex was confirmed in both eyes. For the study of repeatability, the children’s right eyes were examined by the same examiner, who took 3 consecutive measurements. Subsequently, two skilled operators used the AL-Scan randomly in order to evaluate the interobserver reproducibility. Agreement between the AL-Scan and IOLMaster was assessed by the same observer who tool 3 measurements consecutively with every optical biometer. The measurements were conducted between 10:00 a.m. and 5:00 p.m. minimize the diurnal variations of corneal shape and thickness^32,33^. According to the operation manual, each subject was instructed to sit in front of the instrument, place the chin on the chinrest, fix the target, and adjust the position to mark the outer canthus of the patient and the horizontal lines of IOLMaster at the same height. Then, the children were asked to blink before examination to minimize the effect of tear film irregularities. The measurement for each child was completed within 30 min. Only high quality and eligible measurements were selected for further analysis and unreliable data were excluded.

### Intraocular Lens Power Calculation

In order to assess the effect of measurements difference in the clinical setting, we calculated the intraocular lens (IOL) power using the K and AL values from both devices. The SRK/T formula, with an A-constant of 119.0, was chosen for this purpose^34^.

### Statistical Analysis

All data were analyzed using SPSS for Windows software (version 21, IBM Co., USA) and MedCalc statistical software (version 13.0, MedCalc Software Inc., Belgium). A *P*-value < 0.05 was considered statistically significant. Normal distribution was assessed by the Kolmogorov–Smirnov test, and a *P* < 0.05 was considered normally distributed. The results of all parameters were presented as the mean ± standard deviation (SD). The intraobserver repeatability and interobserver reproducibility of the AL-Scan were analyzed using the within-subject SD (S_w_), test-retest repeatability (TRT), within-subject coefficient of variation (CoV), and intraclass correlation coefficients (ICC). The TRT = 2.77S_w_ denoted the interval within which, the 95% of the differences were located. The CoV was calculated as the S_w_ divided by the mean of all measurements; low values revealed better precision. In addition, if the ICCs were closer to 1, the reliability was higher. The paired t-test and Bland–Altman plots with 95% limits of agreement (LoA) were used to evaluate the agreement between the AL-scan and IOLMaster. The 95% LoA was calculated as the average difference between the AL-Scan and IOLMaster ± 1.96SD^35^. The narrower the 95% LoA, the better the agreement.

## Electronic supplementary material

Supplementary Information
