# Supplementary material for: Precision of a new ocular biometer in children and comparison with IOLMaster
Source: Sci Rep. 2018 Jan 22;8:1304. doi: 10.1038/s41598-018-19605-6 (PMC5778016; doi:10.1038/s41598-018-19605-6)
Supplement: Supplementary file 1 — Supplementary Information [file 41598_2018_19605_MOESM1_ESM.doc]

**Precision of a new ocular biometer in children and comparison with IOLMaster**

Xinxin Yu^1^*, MD; Hao Chen^1^*, MD; Giacomo Savini^2^, MD; Qianqian Zheng^1^, MD; Benhao Song^1^, MD; Ruixue Tu^1^, MD; Jinhai Huang^1^, MD, PhD; Qinmei Wang^1^, MD

**Author names, degrees and affiliations:**

^1^ School of Ophthalmology and Eye Hospital, Wenzhou Medical University, Wenzhou, Zhejiang, China.

^2^ G.B. Bietti Foundation IRCCS, Rome, Italy.

**PRÉCIS**

The new biometer (AL-Scan) showed a high repeatability, reproducibility, and consistent measurements and can be used as a common alternative method in children.
